# The asymmetric impact of decision-making confidence on regret and relief

**DOI:** 10.3389/fpsyg.2024.1365743

**Published:** 2024-04-08

**Authors:** Zan Liu

**Affiliations:** School of Education and Music, Sanming University, Sanming, China

**Keywords:** decision-making confidence, counterfactual thinking, regret, relief, action effect

## Abstract

When individuals make uncertain decisions, they often evaluate the correctness of their choices in what is referred to as decision-making confidence. The outcomes of such decision-making can lead to counterfactual thinking wherein alternative possible outcomes are contemplated. This, in turn, can elicit counterfactual emotions including upward and downward counterfactual thinking, which, respectively, refer to regret and relief. Decision-making confidence and counterfactual emotions have key effects on how individuals learn from the past and prepare for the future. However, there has been little understanding of how these experiences are related. For this study, 98 total adults were recruited with the goal of assessing the connections between decision-making confidence and sensations of regret and relief when completing a card-based gambling task. The results of this study suggest that decision-making confidence may reduce the intensity of relief while increasing the degree of regret experienced. These findings thus emphasize the important effect that decision confidence has on emotional processing.

## 1 Introduction

Individuals must inevitably make a large number of decisions each day, and uncertainty in the decision-making process complicates the decision-making process in many cases. After a decision has been made, people often consider the potential outcomes that would have arisen had an alternate decision been made (FitzGibbon et al., 2021). Indeed, such reflection and reconsideration of past decisions is a common facet of human reasoning, and this process of retroactively altering the imagined outcomes of particular events has been termed counterfactual thinking or counterfactual reasoning (Epstude and Roese, 2008; Van Hoeck et al., 2015; Byrne, 2016; Briazu et al., 2017; Huang et al., 2021). Such counterfactual thinking can provide adaptive benefits, enabling individuals to learn from these prior experiences such that they can better prepare for the future (Byrne, 2016; Roese and Epstude, 2017), thus bridging the past and future (Pieters and Zeelenberg, 2007). A range of emotional responses can be evoked by counterfactual thinking, including both regret and relief. Regret is experienced by individuals who engage in upward counterfactual thinking, which entails the imaging of how an alternative decision should have been made and how better outcomes may have been achieved for a given event (Bell, 1982; Landman, 1993; Gilovich et al., 1995; Pieters and Zeelenberg, 2007). Conversely, relief is experienced when the imagined state is worse than the actual situation associated with a given decision or event (Connolly and Zeelenberg, 2002; Liu et al., 2016; De Brigard et al., 2019).

Decision-making confidence refers to an individual’s estimation of the correctness of their choices in an uncertain situation, and such confidence can strongly affect emotional responses to a given situational outcome (Kirkebøen et al., 2013; Kirkebøen and Nordbye, 2017). Confidence is a subjective feeling that refers to one’s belief in the validity of their knowledge, choices, or actions, serving as a measure of the extent to which a given person believes a particular thought or action to be accurate (Yeung and Summerfield, 2012; Grimaldi et al., 2015; Navajas et al., 2016; Dotan et al., 2018). Given that they can evaluate their own decisions in great detail, individuals are often able to recognize mistakes that they have made even when they do not receive any direct feedback. The degree of certainty that individuals express in response to particular choices also varies (Yeung and Summerfield, 2012). In research settings, retrospective judgments are often used to quantify levels of decision-making confidence, with confidence ratings being the most commonly employed approach (Grimaldi et al., 2015). This strategy entails asking participants to rate their degree of confidence from 0% (total uncertainty) to 100% (total certainty). Decision-making confidence and counterfactual thinking are key facets of the decision-making process, and both have important implications for the ability of individuals to learn from particular experiences and for their overall well-being (De Martino et al., 2013; Brus et al., 2021; Liu et al., 2022). The precise link between decision-making confidence and counterfactual thinking, however, remains to be fully characterized.

Several emotion-related theories that have been advanced to date have potential implications for the association between decision-making confidence and counterfactual thinking. The first of these is the subjective expected pleasure theory (SEP; Mellers et al., 1999), which posits that unexpected outcomes tend to evoke emotions stronger than those resulting from expected outcomes. This theory suggests that the magnitude of regret following a poor decision is likely to be reduced if the individual who made that decision had lower decision-making confidence before being aware of the outcome. Conversely, the relief experienced after making an appropriate decision will be reduced if the individual was already highly confident in the decision that they made. An alternative model of the link between confidence and regret can be inferred based on decision justification theory (DJT; Connolly and Zeelenberg, 2002), which suggests that justifications and feelings of self-blame influence the degree of regret. The degree of regret that individuals experience has been shown to be impacted by whether or not they perceive themselves as being responsible for a given mistake. Seemingly unreasonable choices made with lower levels of confidence may thus result in individuals feeling responsible for having made an “incorrect” decision. van de Calseyde et al. (2018) performed six studies based on experimental manipulation and autobiographical recall, and ultimately found that lower levels of confidence in the form of doubts arising following a decision can exacerbate regret through the enhancement of feelings of self-blame after making a poor judgment, in line with the DJT. However, they observed mixed results with respect to how post-decisional doubt relates to the experience of relief. In an incentivized trivia game, similar to the effect on regret, doubting one’s decision before knowing the outcome produced more relief after learning that one’s decision was correct. However, in another incentivized trust game, they did not find any significant relationship between doubt and relief.

The present study was conceptualized with the goal of exploring the association between decision-making confidence and experiences of regret and relief using a card game-based task. In each round of this task, participants were presented with two options: to secure their current gains or to opt for the potential for further gains at the risk of losing their accumulated earnings. Relief and regret were, respectively, experienced when the actual outcome was superior and inferior to the counterfactual outcome. For these analyses, an action effect was hypothesized (Kahneman and Tversky, 1982; Feldman, 2020; Feldman et al., 2020), in which individuals were posited to experience greater regret following negative outcomes arising as a result of action relative to inaction. Many studies have confirmed that acting is associated with greater odds of experiencing regret as compared to a failure to act. Although this action effect has been studied in the context of regret in several prior studies, far less is understood regarding the interplay between this action effect and relief. Accordingly, this study sought to replicate this traditional “regret action effect” while also determining whether it can also be observed in situations characterized by feelings of relief.

## 2 Methods

### 2.1 Participants

This study enrolled 98 undergraduate students (*M*_age_ = 20.88, *SD*_age_ = 1.15; range: 18–28 years; 49 females). All procedures performed in studies involving human participants were following the ethical standards of the institutional and/or national research committee and in accordance with the 1964 Helsinki Declaration and its later amendments or comparable ethical standards. The University’s Ethics Committee approved this study, and all participants provided informed consent.

### 2.2 Procedure

**Figure 1:**
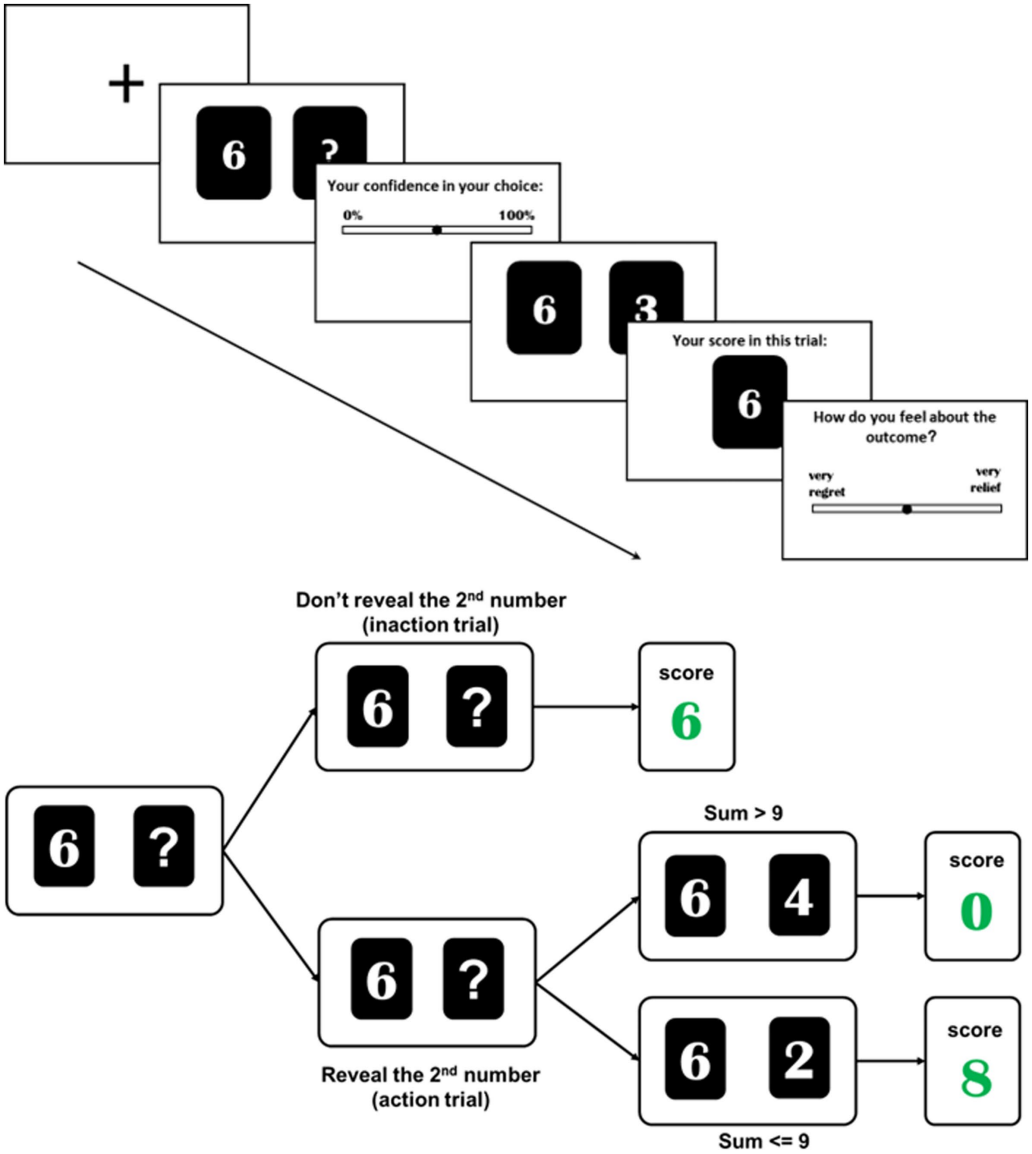
Experimental approach and scoring rules.

To elicit feelings of regret and relief in a counterfactual framework, playing cards were used to establish a gambling task. In each round of testing, two cards were presented on the computer screen. The leftmost card presented the first number, which was a random integer from 0 to 9. Participants were then directed to select whether or not to reveal the second number, which was another random integer from 0 to 9 that was initially hidden on the right card. If participants elected not to reveal the second number (inaction trial), then the first number was their score for that round. If they instead elected to reveal the second number (action trial), their score was equal to the sum of the two numbers provided the total was ≤9, while their score was otherwise 0. The total possible points that a participant could thus earn each round was 9, with a minimum of 0. Participants were directed to try to achieve as high a score as possible in order to obtain a greater reward. The task consisted of 100 total trials, with each combination of numbers appearing only a single time in a randomized order. After making a decision (action vs. inaction), participants were asked to report their level of confidence in the decision that they made on a scale from −50 (low confidence) to +50 (high confidence). The second number was then revealed, and participants were asked to report their feelings of regret or relief on a scale from −50 (extremely regretful) to +50 (extremely relieved). For example, in a trial where the first number was 6 and a participant elected not to reveal the second number, which was subsequently revealed to be a 3, feelings of regret would be expected. In contrast, the participant would instead be expected to experience relief if they elected to reveal the second number and it was subsequently revealed to be a 3. Trials in which the actual and counterfactual outcomes were identical because the second number was 0 were classified as the neutral condition. For further information on the experimental paradigm and the scoring rules, see Figure 1.

The expected values (EVs) for scores when electing to reveal the second card in an action trial are calculated with the following formula:


$$EV=\frac{\left(i+9\right)\left(10-i\right)}{2}*\frac{1}{10}$$

where *i* represents the number on the first card. The EVs for action and inaction trials are (4.5, 0), (4.5, 1), (4.4, 2), (4.2, 3), (3.9, 4), (3.5, 5), (3, 6), (2.4, 7), (1.7, 8), and (0.9, 9). The optimal strategy in this gambling task is thus to only reveal the second number when the first number is 3 or less (see Supplementary Figure 1A). JASP (https://jasp-stats.org/; JASP Team, 2021) was used to conduct all analyses, and the Bonferroni method was used to correct *p* values from follow-up tests.

## 3 Results

### 3.1 Task performance

Average decision-making confidence, decision time, and the ratio of choosing to reveal the second number given each candidate number on the first card were calculated for all study participants (see Supplementary Table 1; Supplementary Figures 1B–D). Using the first number as the independent variable *f*(*x*), a logistic function was fitted (*R*^2^ = 0.99) based on the proportion of participants who elected to reveal the second card.


$$f\left(x\right)=a+\frac{b}{1-e^{-\frac{x-c}{d}}}$$

When *f*(x) equals 0.5, this indicates that the odds of an action and an inaction trial were equal to one another. The value of *x* was 4.57. These analyses revealed that when the first number was 4 or 5, participants exhibited lower levels of decision-making confidence relative to all other experimental conditions, with a corresponding increase in the amount of time required to decide whether to reveal the second number.

Differences between the actual and counterfactual outcomes were defined as the counterfactual difference (CFD). All trials were classified into three conditions: negative (CFD < 0), neutral (CFD = 0), and positive (CFD > 0), and the emotional responses of participants were arranged in a heat-map (see Supplementary Figure 2). In this heat-map, the negative condition accounted for 22% of trials, while the positive condition accounted for 68%, and the neutral condition accounted for 10%.

### 3.2 The relationship between decision-making confidence and feelings of regret and relief

Two Linear Mixed Model (LMM) analyses were conducted to explore the effects of decision-making confidence on regret and relief. In the established LMM, emotions reported by study participants served as the dependent variable, while fixed effects included decision-making confidence and counterfactual difference, both of which were treated as continuous variables. The subject was entered as random intercept (Table 1).

**Table 1:** The impact of decision-making confidence on regret and relief.

	*B*	*SE*	*df*	*t*	*p*
Negative condition					
Intercept	4.60	0.85	87.37	5.40	<0.001
Counterfactual difference	5.43	0.29	96.78	18.72	<0.001
Decision-making confidence	−0.07	0.02	87.09	−2.83	0.006
Positive condition					
Intercept	26.59	1.34	90.74	19.81	<0.001
Counterfactual difference	0.87	0.11	96.76	7.98	<0.001
Decision-making confidence	−0.34	0.03	87.49	−11.32	<0.001

In instances of negative condition, counterfactual difference had a significant influence on the experienced emotion (*B* = 5.43, *SE* = 0.29, *df* = 96.77, *t* = 18.72, *p* < 0.001), indicating that participants experienced higher levels of regret with as the degree to which the counterfactual outcome was better than reality rose. Moreover, decision-making confidence had a significant effect (*B* = − 0.07, *SE* = 0.03, *df* = 87.09, *t* = −2.83, *p* < 0.001). This suggests that, in cases of unfavorable outcomes as a result of a participant’s decision, higher levels of confidence in this decision will result in a higher degree of regret, consistent with the predictions of the SEP. Specifically, the negative outcome of a decision made with greater confidence evoked a stronger emotional response when an unexpected result occurred.

In instances of positive condition, counterfactual difference also had a significant impact on the experienced emotion (*B* = 0.86, *SE* = 0.11, *df* = 96.76, *t* = 7.98, *p* < 0.001). This indicates that as the real outcome improved as compared to the counterfactual outcome, participants experienced a higher degree of relief. The effect of decision-making confidence was also significant (*B* = −0.34, *SE* = 0.03, *df* = 87.09, *t* = −11.32, and *p* < 0.001). When a decision made by a participant resulted in a positive outcome, greater decision-making confidence was thus associated with a reduction in the level of relief experienced.

### 3.3 Emotional differences among the three conditions

Differences in emotions among the three different conditions were next tested using a Linear Mixed Model (LMM) analysis. In this analysis, the dependent variable was the emotion that participants reported experiencing, while fixed effects included the choice made (action vs. inaction) and the outcome condition (positive, neutral, and negative), which were both categorical variables. The subject was entered as random intercept.

**Figure 2:**
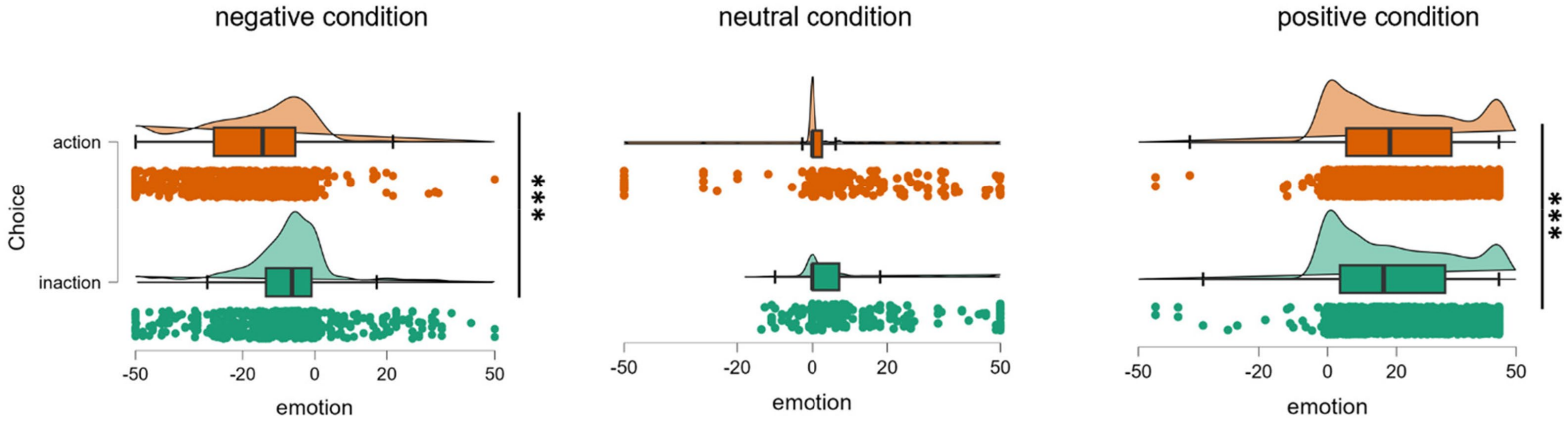
Emotional differences among different conditions. Two-way ANOVA results: 2 (choice: action & inaction) × 3 (outcome condition: negative, neutral, and positive) (The Raincloud plots are made up of three parts, namely density estimate plot, box plot, and dithering scatter plot).

These analyses revealed that the main effects of condition and choice were both significant [*F*(96.68, 2) = 248.73, *p* < 0.001; *F*(94.12, 1) = 39.97, *p* < 0.001], as was the interaction effect [*F*(97.21, 2) = 51.04, *p* < 0.001]. A simple effect analysis indicated that action was associated with higher levels of regret in the negative condition (*t* = −8.76, *p*_bonferroni_ < 0.001), while also being associated with stronger feelings of relief in the positive condition (*t* = 3.24, *p*_bonferroni_ = 0.004). In the neutral condition, there was no significant difference between action and inaction (*t* = 2.80, *p*_bonferroni_ = 0.076) (Figure 2).

## 4 Discussion

Confidence and counterfactual thinking are key facets of the decision-making process. The results of the present study revealed that higher levels of confidence were sufficient to reduce experiences of relief while increasing experiences of regret among participants completing a gambling task. Per the SEP theory, unexpected results can provoke more intense emotions than expected outcomes. When an individual has total confidence in a given decision and the outcome aligns with these expectations, they will experience lower levels of relief. Conversely, if they have high levels of confidence but then experience an unexpected adverse outcome, they are likely to experience a greater sense of regret. These analyses also replicated the well-characterized action effect of regret, while also demonstrating that this action effect also extends to experiences of relief. Specifically, in the negative condition, action was associated with the worsening of participant emotions (regret), whereas in the positive condition, it was associated with the enhancement of these emotions (relief).

Counterfactual thinking necessitates that an individual process both the true and counterfactual realities (Redshaw and Suddendorf, 2020; Allaert et al., 2021; Tagini et al., 2021). The difference between these two realities influences the intensity of regret or relief experienced by an individual, with this comparison providing a premise to experience counterfactual emotions. Individuals who exhibit a high degree of confidence regarding their choice believe that their expected outcome will ultimately match the true outcome. In an eye-tracking study using the Wheel of Fortune task, researchers confirmed that participants compared their outcomes and the unselected lottery outcomes during the feedback phase in the full feedback condition, particularly following losses (Bault et al., 2016). This highlights an asymmetry with respect to experiences of upward counterfactual thinking (regret) and downward counterfactual thinking (relief), with negative outcomes being more likely to give rise to counterfactual thinking. Individual expectations may underlie this phenomenon. Under the relief condition, true outcomes were consistent with expectations. In contrast, under the regret condition, expectations and the aversive true outcome differed, prompting a comparison of reality and counterfactual realities that would result in experiences of counterfactual emotions in the form of regret. Decision-making confidence may amplify such asymmetry, given that confidence reflects a given individual’s belief that their decision is accurate (Pouget et al., 2016; Brus et al., 2021). Here, decision-making confidence was found to reduce relief (downward counterfactual thinking) while enhancing regret (upward counterfactual thinking).

In the first report demonstrating the action effect (Kahneman and Tversky, 1982), participants were asked to compare two investors who had initially agreed to invest in company A, one of whom ultimately elected to take action by switching their investments to company B, whereas the other took no action. Both investors ultimately lost an equal amount of money. Study participants attributed greater regret to the investor who took action and switched their investments. Since this initial description, the action effect has been replicated in many studies with a focus on the experience of regret (Towers et al., 2016; van de Calseyde et al., 2018; Feldman, 2020; Jamison et al., 2020). In the present study, this action effect was found to be intact under conditions of both relief and regret. Action has been attributed to stronger links between behaviors and outcomes and a greater sense of responsibility for negative outcomes relative to inaction (Jamison et al., 2020). This link between action and responsibility can reinforce feelings of self-blame and contribute to greater levels of regret. A similar process was herein observed under the relief condition, with action contributing to greater relief, possibly as a result of stronger self-attribution, thus serving a self-promoting function.

Together, these data suggest that confidence can abrogate relief while increasing levels of regret, while also demonstrating that the traditional action effect is present in scenarios characterized by both regret and relief. This underscores the importance of meta-cognitive decision-making-related processing for a given individual’s perceptions of outcomes following a given decision, while also offering further insight into the processes that shape risk-taking behaviors in uncertain situations.

## Data availability statement

The raw data supporting the conclusions of this article will be made available by the authors, without undue reservation.

## Ethics statement

The studies involving humans were approved by Ethics Committee of Sanming University. The studies were conducted in accordance with the local legislation and institutional requirements. The participants provided their written informed consent to participate in this study.

## Author contributions

ZL: Writing – review & editing, Writing – original draft.
