# Supplementary material for: The asymmetric impact of decision-making confidence on regret and relief
Source: Front Psychol. 2024 Apr 8;15:1365743. doi: 10.3389/fpsyg.2024.1365743 (PMC11033447; doi:10.3389/fpsyg.2024.1365743)
Supplement: Supplementary file 1 [file Table_1.DOCX]

**Supplementary Material**


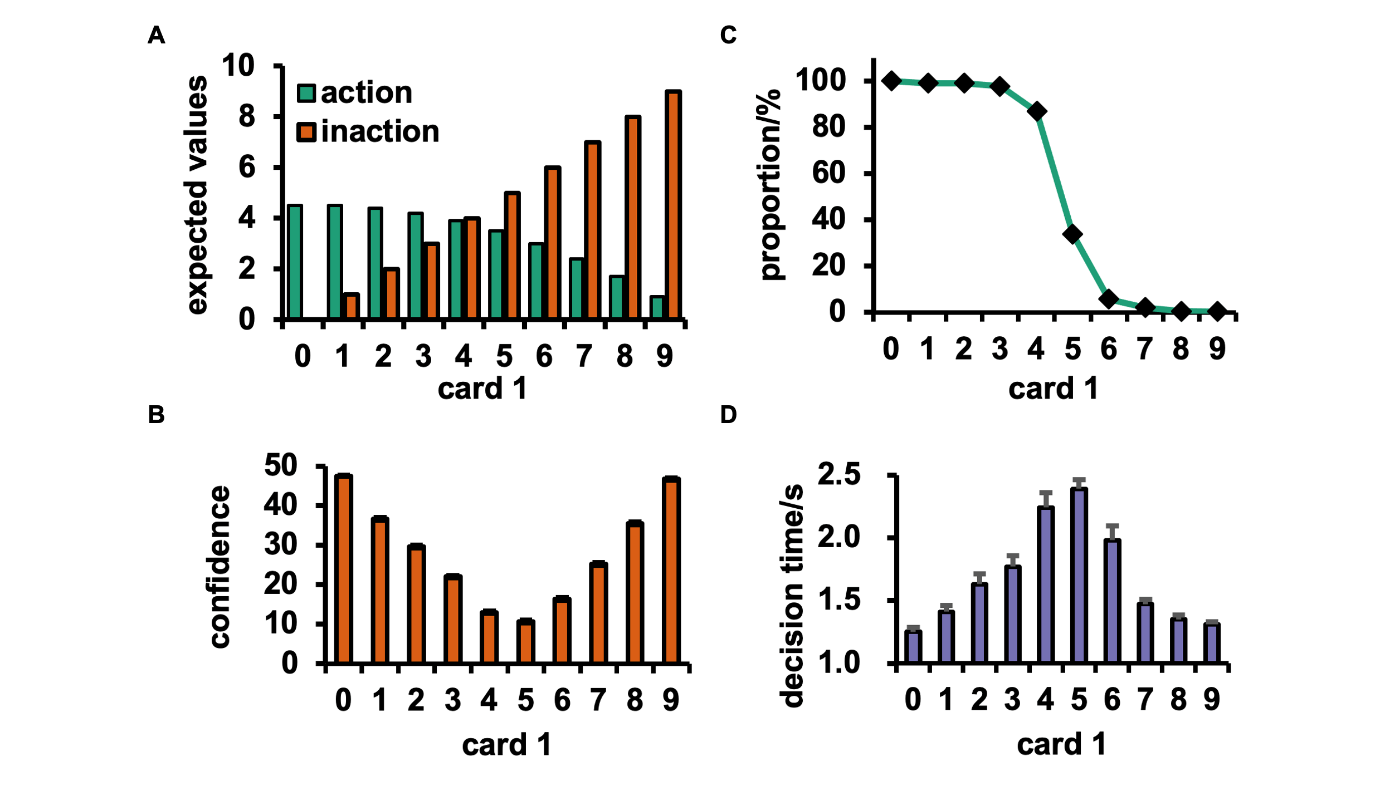


Figure 1 Task Performance A. Expected values for action and inaction trials. B. The actual proportions of choosing to reveal the second card for each number on the first card. C: Average confidence levels for each number on the first card. D. Average decision times for each number on the first card.

Table 1 Descriptive statistics corresponding to confidence, decision time, and the proportions of participants electing to reveal the second card for each number on the first card.

|  |  | The number on the first card | | | | | | | | | |
| --- | --- | --- | --- | --- | --- | --- | --- | --- | --- | --- | --- |
|  |  | 0 | 1 | 2 | 3 | 4 | 5 | 6 | 7 | 8 | 9 |
| confidence | *M* | 47.5 | 36.6 | 29.6 | 22 | 13 | 10.7 | 16.4 | 25.2 | 35.5 | 46.7 |
|  | *SE* | 0.271 | 0.416 | 0.429 | 0.407 | 0.396 | 0.424 | 0.442 | 0.434 | 0.44 | 0.348 |
| decision time | *M* | 1.25 | 1.41 | 1.63 | 1.77 | 2.24 | 2.39 | 1.98 | 1.47 | 1.35 | 1.31 |
|  | *SE* | 0.034 | 0.048 | 0.081 | 0.084 | 0.120 | 0.070 | 0.115 | 0.039 | 0.035 | 0.022 |
| proportion | *M* | 100 | 99 | 99 | 97.6 | 86.8 | 34 | 5.6 | 1.9 | 0.4 | 0.2 |
|  | *SE* | 0 | 0.316 | 0.33 | 0.521 | 1.21 | 1.69 | 0.788 | 0.452 | 0.211 | 0.145 |

Based on the observed decision-making behavior results, when the first number was greater than 4, very few participants elected to reveal the second number. The bottom half of the established heat-map thus corresponds to inaction trials, whereas the upper half corresponds to action trials for which the first number was less than 5 such that most participants elected to reveal the second number, and the top half of the heat-map was based on “action” trials.


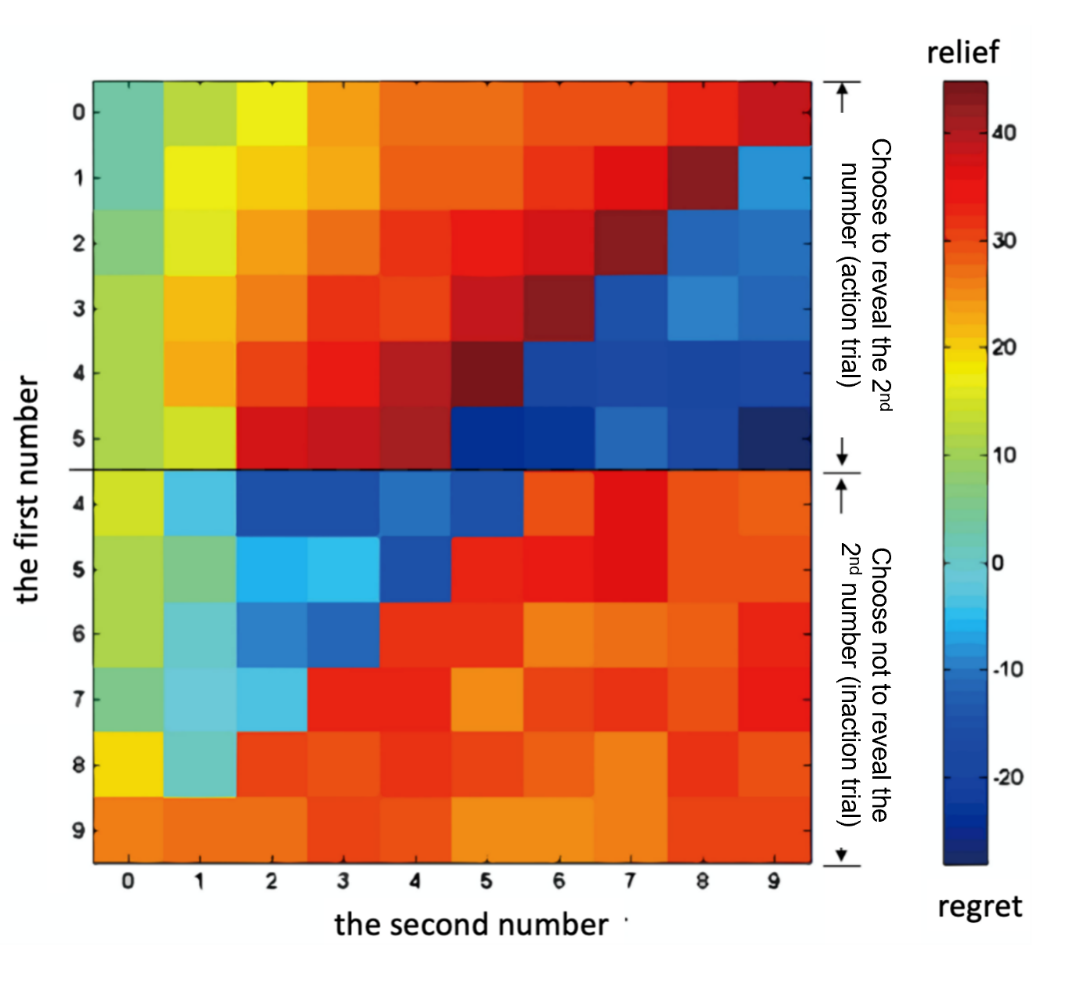


Figure 2 Participants’ emotional responses. The upper portion of the figure represents the emotional responses of participants when opting to reveal the second number (action trials). When the first number is 5 and the participant elects to reveal the second number, which is also 5, for example, the final score will be 0 and they will experience a very high degree of regret. The lower portion of the figure represents the emotional responses of participants when they do not opt to reveal the second number (inaction trials).
